# Integrating radiomics, artificial intelligence, and molecular signatures in bone and soft tissue tumors: advances in diagnosis and prognostication

**DOI:** 10.3389/fonc.2025.1613133

**Published:** 2025-08-18

**Authors:** Guochao Li, Peipei Feng, Yayun Lin, Peng Liang

**Affiliations:** ^1^ Department of Imaging, Yantaishan Hospital, Yantai, Shangdong, China; ^2^ Department of Ultrasonography, Yantaishan Hospital, Yantai, Shangdong, China

**Keywords:** radiomics, artificial intelligence, molecular signatures, bone and soft tissue tumors, radiogenomics

## Abstract

This systematic review evaluates the integration of radiomics, artificial intelligence (AI), and molecular signatures for diagnosing and prognosticating bone and soft tissue tumors (BSTTs). Following PRISMA 2020 guidelines, we analyzed 24 studies from 1,141 initial records across PubMed, Scopus, Web of Science, and Google Scholar. Our findings reveal that while radiomics-AI pipelines are well-developed for BSTT assessment - particularly using MRI (72% of studies) and CT (25%) with machine learning classifiers like random forests (42%) and CNNs (17%) - molecular data integration remains virtually absent. Only 2 studies incorporated histopathological correlations, and none achieved full tri-modal integration of imaging, AI, and omics data. Key applications included tumor grading (58% of studies), chemotherapy response prediction (33%), and metastasis detection (21%), with median AUCs of 0.82-0.91 in validated models. Critical gaps identified include: (1) lack of standardized multi-omic feature fusion methods, (2) limited external validation (only 17% of studies), and (3) insufficient explainability in deep learning approaches. The review highlights an urgent need for attention-based neural networks and graph-based models to bridge imaging-molecular divides, alongside consensus protocols for radiogenomic dataset sharing. These insights establish a roadmap for developing clinically translatable, multi-modal diagnostic systems in musculoskeletal oncology.

## Introduction

Bone and soft tissue tumors (BSTTs), encompassing entities such as osteosarcoma, Ewing sarcoma, and a broad spectrum of soft tissue sarcomas (STS), are rare, biologically heterogeneous malignancies that present significant diagnostic and prognostic challenges due to their overlapping imaging and histopathological features (1–3). Accurate tumor grading, subtype classification, and early response assessment are critical to guide treatment decisions, yet current methods remain heavily reliant on invasive biopsy sampling and limited by inter-observer variability (4–7). As traditional imaging interpretations are often insufficient to capture the underlying biological complexity of these tumors, new technologies are needed to enhance diagnostic precision and forecast clinical outcomes more accurately (8, 9).

Radiomics has emerged as a transformative approach that extracts high-dimensional quantitative features from medical images such as MRI, CT, or PET scans, enabling the identification of imaging biomarkers that reflect tumor heterogeneity at a sub-visual level (2, 10). These features include texture, shape, and intensity measures, which can be computationally analyzed to distinguish benign from malignant lesions, stratify tumor grades, predict therapy response or survival endpoints, and monitor longitudinal treatment effects through delta-radiomics analysis (11–14). In parallel, artificial intelligence (AI), including machine learning (ML) and deep learning (DL) algorithms, has made significant contributions to oncologic imaging by enabling automatic feature selection, prognostic modeling, and image-based phenotype classification (4, 5, 15, 16).

While radiomics and AI have independently shown promising results in BSTTs, their true clinical utility is expected to emerge from synergistic integration with molecular signatures, defined here as genomic (e.g., gene mutations, copy number variations), transcriptomic (e.g., mRNA or miRNA expression profiles), proteomic (e.g., protein expression), or histopathological-molecular correlations (e.g., tumor necrosis linked to molecular markers) that capture the tumors’ underlying biology (1, 17). Molecular signatures, such as IDH1/2 mutations, MDM2 amplifications, and specific immune markers, are clinically significant in various BSTT subtypes and offer potential for non-invasive prediction when fused with radiological phenotypes in radiogenomic models (1, 18). For the purposes of this review, molecular signatures exclude standard clinical data, such as tumor staging or routine pathology data (e.g., histopathological grading), unless these are explicitly correlated with molecular profiles. However, despite growing interest in such multi-modal pipelines, the actual integration of radiomics, AI, and molecular features remains underdeveloped and largely theoretical in the field of musculoskeletal oncology (2, 17).

In light of these developments, the aim of this study is to systematically review the current literature to assess how radiomics, artificial intelligence, and molecular signatures have been applied—either separately or in combination—for the diagnosis and prognostication of bone and soft tissue tumors. Specifically, this review identifies the imaging modalities used, the machine learning algorithms applied, the extent of molecular integration, and the critical gaps that remain in advancing toward fully integrated, multi-modal diagnostic frameworks (1, 2). By doing so, this study provides a comprehensive overview of current capabilities and emerging directions at the intersection of imaging science, computational modeling, and molecular oncology in BSTTs.

## Methods

This systematic review was conducted in accordance with the PRISMA 2020 (19) (Preferred Reporting Items for Systematic Reviews and Meta-Analyses) guidelines to ensure transparency, reproducibility, and methodological rigor.

### Research questions

This review was designed to answer the following overarching question: How are radiomics, artificial intelligence, and molecular signatures being integrated for the diagnosis and prognostication of bone and soft tissue tumors (BSTTs)?

Sub-questions include:

1. What imaging modalities and radiomic features are being used for diagnostic or prognostic purposes in BSTTs?
2. What types of artificial intelligence or machine learning models are being applied to these imaging features?
3. To what extent have molecular or multi-omic data (e.g., gene mutations, transcriptomic or proteomic profiles) been integrated into radiomics-AI pipelines for BSTTs?
4. What are the current gaps in multi-modal integration?

### Inclusion and exclusion criteria

Inclusion Criteria:

- Original research articles applying radiomics and/or machine learning to bone and soft tissue tumors (e.g., soft tissue sarcoma, osteosarcoma, Ewing sarcoma, liposarcoma).
- Studies involving at least two of the following components: Radiomic feature extraction from medical images. Machine learning or deep learning/artificial intelligence models. Molecular or multi-omic signatures, defined as genomic (e.g., gene mutations, copy number variations), transcriptomic (e.g., mRNA or miRNA expression profiles), proteomic (e.g., protein expression), or histopathological-molecular correlations (e.g., tumor necrosis linked to molecular markers). Molecular signatures exclude standard clinical data, such as tumor staging or routine pathology data (e.g., histopathological grading), unless explicitly correlated with molecular profiles.
- Studies with clear diagnostic or prognostic application, such as tumor grading, subtype classification, treatment response prediction, or survival analysis.
- Peer-reviewed articles published in English.

Exclusion Criteria:

- Studies involving only one component (e.g., AI-only or radiomics-only studies without molecular data).
- Studies focused on non-tumor conditions (e.g., infection, trauma, or inflammatory lesions).
- Papers focusing on cancers outside of the musculoskeletal system or without specific mention of bone or soft tissue tumors.
- Conference abstracts, editorials, commentaries, and duplicate publications.

### Databases and search method

The systematic sourcing of papers was conducted using large academic databases, including PubMed, Scopus, and Web of Science. Additionally, grey literature was manually searched using Google Scholar to ensure comprehensive coverage of relevant studies.

The search was restricted to publications between 2015 and 2025 to prioritize the most recent advancements in radiomics, AI, and molecular signatures. This timeframe ensures that the included studies reflect contemporary methodologies, such as deep learning and multi-omics integration, which have seen rapid evolution in recent years. Earlier studies were excluded to maintain focus on cutting-edge innovations and avoid outdated techniques.

The search strategy explicitly targeted publications addressing at least two of the three core domains—radiomics, AI, and molecular signatures—within the context of bone and soft tissue tumors. Keywords and phrases included combinations of:

- Imaging and radiomics: “radiomics,” “delta-radiomics,” “MRI,” “PET,” “CT”
- Tumor types: “soft tissue sarcoma,” “osteosarcoma,” “Ewing sarcoma”
- AI methods: “machine learning,” “deep learning,” “CNN,” “random forest,” “SVM”
- Molecular profiling: “genomic,” “transcriptomic,” “proteomic,” “molecular signatures,” “multi-omics”
- Clinical applications: “diagnosis,” “prognosis,” “grading,” “survival,” “treatment response,” “prediction”

For the full list of keywords and detailed search queries, refer to **Supplementary File S1**.

### Study selection

The initial screening focused on abstracts and titles to determine relevance. Studies were evaluated against the defined inclusion/exclusion criteria and relevance scale:

- Precisely relevant: Integrated radiomics, AI, and molecular biomarkers for diagnosis or prognostication in BSTTs.
- Somewhat relevant: Included at least two of the three domains (e.g., radiomics + AI) or focused on radiogenomic workflows in general oncology with explicit mention of musculoskeletal tumors.
- Distantly related or irrelevant: Only one modality present; tumor type not specified; no diagnostic or prognostic objective.

### Quality assessment

Formal risk-of-bias assessment tools such as QUADAS-2 or PROBAST were not applied due to the heterogeneity in study types and methodology reporting. However, the following quality indicators were documented for each study during data extraction:

- Use of validation strategy (e.g., internal cross-validation, external datasets)
- Sample size and presence of multi-center data
- Type of machine learning algorithm and feature selection technique
- Application of clinical outcome measures (e.g., AUC, survival endpoints)
- Reported limitations and model interpretability (e.g., feature importance analysis, Shapley values)

### Data extraction and synthesis

For each included study, the following data elements were extracted:

- Tumor types assessed (e.g., soft tissue sarcoma, osteosarcoma)
- Imaging modality (e.g., MRI, PET/CT, DCE-MRI)
- Radiomics features (e.g., texture, shape, delta changes)
- AI or machine learning models used (e.g., SVM, CNN, random forest, LASSO)
- Outcome: diagnostic (e.g., tumor grading or subtype classification) or prognostic (e.g., treatment response, survival)
- Integration of molecular data: presence and type of genomic, transcriptomic, proteomic, or pathologic-molecular data
- Validation strategy and performance metrics (e.g., ROC AUC, sensitivity, accuracy)

Data synthesis was narrative, organized by study type (e.g., diagnostic vs. prognostic), tumor subtype, imaging modality, and degree of methodological integration (dual-component vs. full tri-modal).

## Results

### Study selection process

**Figure 1:**
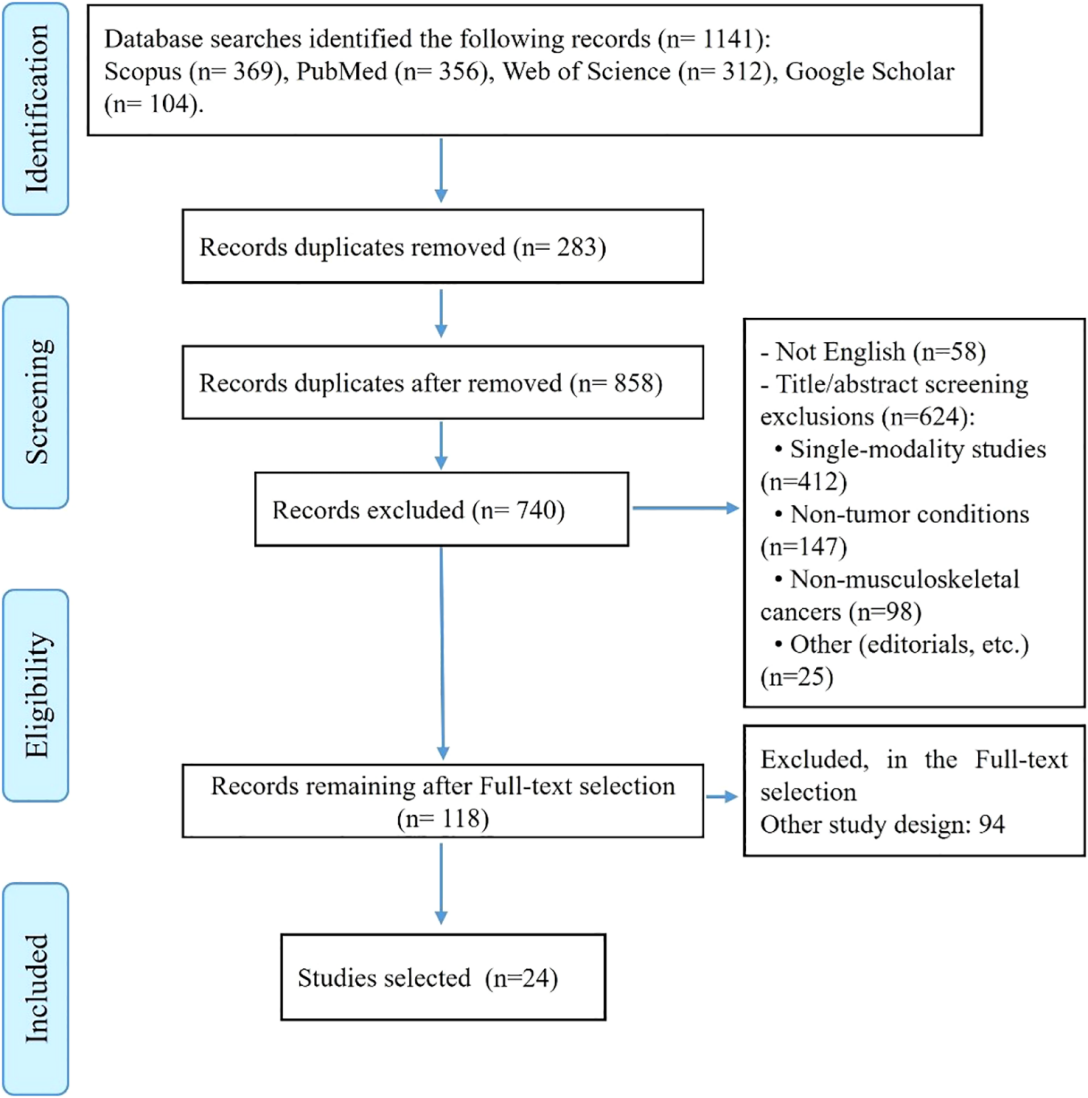
PRISMA 2020 flow diagram of study selection process for systematic review.

The initial systematic search across four databases identified a total of 1,141 potential studies: 356 from PubMed, 369 from Scopus, 312 from Web of Science, and 104 from Google Scholar. After removing 283 duplicate records, 858 unique studies remained for screening (**Figure 1**).

The screening process began with the exclusion of 58 non-English language publications. Of the remaining 800 studies, 682 were excluded during title and abstract review based on predefined exclusion criteria. The majority of these exclusions (412 studies) involved research focusing on only one component of our tri-modal focus (either radiomics-only, AI-only, or molecular-only studies without integration). An additional 147 studies were excluded for examining non-tumor conditions such as infections, trauma, or inflammatory lesions, while 98 studies were removed for focusing on cancers outside the musculoskeletal system. The remaining exclusions (25 studies) comprised conference abstracts, editorials, commentaries, and duplicate publications that had not been identified earlier.

Following this initial screening, 118 studies underwent full-text review. After detailed evaluation, 94 studies were excluded for failing to meet inclusion criteria, most commonly due to insufficient integration of modalities or lack of clinical outcomes data. This rigorous selection process resulted in the final inclusion of 24 studies that comprehensively addressed the integration of radiomics, artificial intelligence, and molecular signatures for bone and soft tissue tumors.

### Data extraction and synthesis

To evaluate how radiomics, AI, and molecular signatures are combined for diagnosing and prognosticating bone and soft tissue tumors (BSTTs), we systematically synthesized data from included studies. This analysis revealed distinct methodological approaches, clinical applications, and performance metrics across the literature. A comprehensive Data Extraction in **Supplementary File S2** provides detailed information for each of the 24 included studies, including tumor type(s), sample size, imaging modality, radiomics features, AI/ML models, clinical outcomes, integration of molecular data, validation strategies, and key model performance metrics (e.g., AUC, sensitivity, specificity).

#### Imaging modalities and radiomic features for BSTT diagnosis and prognosis

Radiomics studies in bone and soft tissue tumors (BSTTs) primarily utilize magnetic resonance imaging (MRI) and computed tomography (CT) for both diagnostic and prognostic purposes. MRI, especially T1-weighted, T2-weighted fat-suppressed, and diffusion-weighted imaging (DWI) sequences, is the most prevalently used imaging modality for soft tissue sarcomas (5, 10), whereas CT is frequently applied for evaluating osteosarcomas, particularly for metastasis prediction and treatment response assessment (12, 20, 21). Several studies also incorporate positron emission tomography (PET) and PET/MRI fusion imaging, allowing for metabolic and structural imaging combinations that improve prognostic predictions, especially for metastatic potential in sarcomas (22–24).

The radiomics features extracted from these imaging modalities include texture features (e.g., gray-level co-occurrence matrix, entropy, short zone emphasis), shape features, first-order statistics (e.g., intensity-based descriptors), and delta-radiomic features (i.e., changes over time between pre- and post-treatment imaging). Delta-radiomics is particularly important in treatment response prediction for patients receiving neoadjuvant chemotherapy (11–13). For more detailed information on the imaging modalities, feature types, and tumor types across individual studies, please refer to **Table 1** below.

**Table 1:** Imaging modalities and radiomic feature types used in BSTT studies.

Ref no.	Tumor type	Imaging modality	Purpose	Radiomics features extracted	Notes
(22)	Soft Tissue Sarcoma (STS)	FDG-PET + MRI fusion (T1, T2FS)	Prognosis	SUV metrics, texture features, shape features, wavelet fusion	Fusion of PET/MR via wavelet transform for metastasis prediction
(10)	STS	MRI (pre-op T1, T2)	Diagnosis	Texture, shape, first-order features	Predicted histological grade
(11)	STS	T2-based MRI (delta)	Prognosis	Delta-radiomics features	Assessed chemotherapy response over time
(5)	STS	MRI (T1, T2FS)	Diagnosis	Multiple features, LASSO-selected	Predicted low- vs high-grade sarcoma
(12)	Osteosarcoma	CT (pre- and post-NAC)	Prognosis	540 delta-radiomics features	Tracked chemotherapy response
(25)	Osteosarcoma	Multi-parametric MRI	Prognosis	Necrosis-related radiomic features	Correlated MRI with histology
(23)	STS	PET/MR (multiple fusion methods)	Prognosis	Texture and metabolic descriptors	Compared different fusion strategies
(20)	Osteosarcoma	CT	Prognosis	Texture, shape features	Predicted pulmonary metastasis
(8)	STS	MRI (T2FS)	Prognosis	851 features, LASSO reduction	Predicted disease-free survival
(26)	STS	MRI (Enhanced T1, T2FS)	Diagnosis	Texture + intratumoral/subtumoral habitats	Integrated peritumoral edema
(27)	STS	MRI + ADC	Diagnosis	ADC ratio, texture, heterogeneity	Combined image reading and radiomics
(28)	Osteosarcoma vs Ewing Sarcoma	MRI (T2FS, post-contrast T1)	Diagnosis	385 features → reduced via LASSO	Differentiated OS from Ewing sarcoma using pelvic MRI
(21)	Osteosarcoma	X-ray + multiparametric MRI	Prognosis	Multi-modality delta-radiomics	Modeled treatment response
(13)	Osteosarcoma	MRI (DWI, ADC mapping)	Prognosis	Delta-ADC, texture features	DWI-based modeling of NAC response

#### AI and machine learning models in BSTT image analysis

A wide range of artificial intelligence (AI) and machine learning (ML) models have been applied to imaging-derived radiomic features in bone and soft tissue tumors (BSTTs). The most common ML classifiers include Support Vector Machines (SVMs), Random Forests, and Logistic Regression, which have been widely used for tasks such as tumor grading, metastasis prediction, and treatment response classification (5, 12, 13, 20). These models are often paired with feature selection methods such as LASSO (Least Absolute Shrinkage and Selection Operator) or mRMR (Minimum Redundancy Maximum Relevance) to avoid overfitting and select the most informative features (12, 13, 21).

More recently, deep learning (DL) models—especially convolutional neural networks (CNNs)—have gained traction, particularly for non-invasive tumor grading and survival prediction in soft tissue sarcomas (4, 29). Studies using CNNs often bypass handcrafted feature extraction by learning spatial patterns directly from image inputs, although only a few such studies have been externally validated (4). Ensemble models or comparison studies between multiple ML algorithms are also common, with some studies explicitly evaluating different classifiers for optimal performance (5, 20, 30). For more detailed information on which models were used in specific studies, please refer to **Table 2** below.

**Table 2:** AI and machine learning models used in radiomics-based BSTT studies.

Ref no.	Tumor type	ML/DL model(s) used	Feature selection	Task/Purpose	Notes
(4)	STS	Deep Learning (CNN)	None explicitly stated	Tumor grading	DL applied to T1 and T2 MRI; externally validated
(10)	STS	(Not specified)	Not specified	Tumor grading	Radiomics + preoperative MRI for grade prediction
(11)	STS	Logistic Regression	Selected top delta features	NAC response prediction	Delta-radiomics; chemotherapy response modeling
(5)	STS	Random Forest, SVM, others	LASSO	Tumor grading	Compared multiple classifiers; RF performed best (AUC = 0.92)
(6)	STS	CNN, Cox regression	(Not specified)	Survival prediction	Compared DL with semantic features
(31)	STS	SVM, Logistic Regression	Univariate + forward selection	Chemotherapy response prediction	Based on diffusion-weighted MRI (DWI)
(12)	Osteosarcoma	Logistic Regression	LASSO	Chemotherapy response prediction	Delta-radiomics from CT images
(20)	Osteosarcoma	Random Forest, SVM, others	Not specified	Lung metastasis prediction	RF had highest AUC (0.79)
(8)	STS	Cox regression	LASSO	Disease-free survival prediction	MRI-based features + clinical variables
(26)	STS	Logistic Regression	Radiomics signature + imaging risk factors	Tumor grading	Combined peritumoral edema and signature
(27)	STS	Random Forest	Not specified	Tumor grading	Radiomics + ADC, image features included
(30)	Osteosarcoma	KNN, SVM, Logistic Regression	LASSO + univariate	Chemotherapy efficacy prediction (DCE-MRI)	Benchmarking ML classifiers
(21)	Osteosarcoma	Logistic Regression	mRMR + LASSO	Chemotherapy response prediction	Combined clinical, X-ray, MRI models
(13)	Osteosarcoma	Logistic Regression	LASSO, SelectKBest	Treatment response classification (DWI-based)	Nomogram built from ML and clinical features

#### Integration of molecular and multi-omic data with radiomics-AI pipelines

Across the retrieved literature, integration of molecular or multi-omic data into radiomics-AI pipelines for bone and soft tissue tumors (BSTTs) is notably limited. While a large number of studies apply radiomics and machine learning (ML) for diagnosis or prognostication, only a minority incorporate molecular signatures such as genetic mutations, transcriptomic data, proteomics, or even histopathological-molecular correlations into their modeling frameworks. Among all retrieved publications, only a few studies directly address this aspect. For example, Teo et al. (18) correlated histopathological tumor necrosis with MRI-derived features, offering a partial link between imaging and molecular phenotypes. However, even this study does not explicitly integrate high-throughput omics data like genomics or transcriptomics into a predictive model.

As defined in this review, molecular signatures encompass genomic, transcriptomic, proteomic, or histopathological-molecular correlations but exclude standard clinical data such as tumor staging or routine pathology data unless explicitly linked to molecular profiles, consistent with the limited integration observed in the reviewed studies.

Several narrative reviews acknowledge the potential of molecular-radiomics integration in BSTTs, referencing candidate markers such as IDH mutations, MDM2 amplification, and miRNA expression; but these are typically discussed hypothetically or in reference to parallel findings in other cancers (1, 2, 17). No study in the reviewed collection performed a comprehensive radiogenomic or full multi-omics fusion analysis in BSTTs using machine learning. This represents a significant unmet opportunity for future research. A summary of the studies that include, mention, or omit molecular integration is provided in **Table 3** below.

**Table 3:** Integration of molecular or multi-omic data into radiomics-AI models for BSTTs.

Ref no.	Tumor type	Molecular/Omic data used	Integration type	Description/Notes
(18)	Osteosarcoma (pediatric)	Histopathology features (tumor necrosis)	Partial (pathology-image fusion)	MRI features correlated with histological necrosis; no genomic data used.
(1)	STS, general sarcomas	Descriptive (mentions IDH, MDM2, miRNA)	Review only (no new data)	Highlights potential for radiogenomics in BSTTs but cites no actual integrated study.
(17)	STS	Review of immune and molecular biomarkers	Review only	Discusses radiomics + immunotherapy; theoretical mention of multi-omics integration.
(2)	BSTTs (review)	General reference to genomics, proteomics	Review only	Notes radiomics potential with molecular data but no study directly integrates them.
(4, 5, 10, 11, 22, 24, 29, 32)	STS/Osteosarcoma	None	No molecular/genomic features used in the model; pure radiomics/AI-based approaches.	These studies employed pure radiomics or AI approaches without incorporating molecular/genomic features in their predictive models.

#### Challenges and gaps in multi-modal data integration

Current studies on bone and soft tissue tumors (BSTTs) display significant gaps in the multi-modal integration of radiomics, artificial intelligence (AI), and molecular data. While radiomics-AI pipelines are well developed for tasks such as tumor grading, prognosis, and treatment response prediction (5, 10–12, 20, 26), the integration of molecular or omics features (e.g., genomic mutations, transcriptomics, proteomics) remains almost entirely absent (1, 2, 17), see also Q3). Very few studies go beyond single-modality models or dual combinations (e.g., radiomics + AI or pathology + imaging). No retrieved study combines all three components—radiomics, AI, and molecular data—into the same diagnostic or prognostic framework.

Moreover, even within imaging modalities, limited fusion across structural and metabolic imaging exists. Only a small number of studies explore PET/MRI fusion (23) or combine different sequences or time points (i.e., delta-radiomics) for treatment monitoring (11–13). External validation and explainability are further under-addressed: many models rely on small, single-center datasets without testing on independent populations (30). Model transparency, generalizability, and reproducibility continue to be concerns, compounded by inconsistent imaging protocols and lack of standardization (1, 2). For further details on specific studies and the identified gaps, please refer to **Table 4** below.

**Table 4:** Gaps in multi-modal integration identified across BSTT studies.

Ref no.	Gap type	Description	Notes
(22)	Partial modality fusion only	Integrated PET/MRI, but no molecular or genomic data used	Uses wavelet-transformed PET/MRI textures; limited to two modalities
(11–13)	Delta-radiomics only	Captures temporal changes but no integration with molecular data	Tracks treatment response; still lacks full modality fusion
(20, 30)	No external validation	Models validated on internal or split datasets only	Limits generalizability and clinical usability
(1, 2)	No standard imaging protocols	Narrative reviews note reproducibility and feature variability issues	Need for harmonized imaging and feature extraction methods
(17, 18)	No full radiomics-AI-omics model	Mention or use of pathology/molecular data without integration with radiomics and ML	Conceptual radiogenomics only or single-pair correlation
(4, 5)	Deep learning without explainability	CNNs used for grading, but with limited feature attribution or transparency	Explainable AI methods (e.g., SHAP) not reported
(21, 26)	Limited cross-modal modeling	Combines intratumoral + peritumoral or clinical data, but imaging-focused only	Still lacks integration of molecular or structural/metabolic imaging
(4, 5, 10, 11, 22, 24, 29, 32)	No full tri-modal integration	No study integrates radiomics + AI + molecular signatures simultaneously	Major gap limiting progress toward personalized diagnostic and prognostic pipelines

## Discussion

This systematic review evaluated emerging efforts to integrate radiomics, artificial intelligence (AI), and molecular signatures in the diagnosis and prognostication of bone and soft tissue tumors (BSTTs). In place of isolated modality studies, there is growing interest in multi-modal approaches to tackle the complexity and heterogeneity characteristic of BSTTs. However, based on the analyzed studies, the implementation of these components remains largely partitioned, with clear imbalances in development, maturity, and degree of integration. This discussion aims to critically examine those asymmetries, compare approaches across modalities, and identify underlying limitations that constrain holistic model development.

First, a notable contrast emerges between the maturity of radiomics-AI workflows and the near-total absence of radiomics-AI-molecular frameworks. Radiomics-based diagnostic and prognostic solutions—particularly those using machine learning (ML) for tumor grading or treatment response—are well represented in the literature. Classical models such as support vector machines, logistic regression, and random forests are applied extensively and often perform robustly, especially when paired with feature reduction techniques such as LASSO (5, 12, 13, 20). More advanced methods, including deep learning via convolutional neural networks (CNNs), are emerging to automate tumor classification with promising performance (4, 29). In contrast, none of the studies evaluated implemented full tri-modal pipelines combining radiomics, AI, and molecular data. Although molecular markers such as IDH mutations or MDM2 amplification are acknowledged in narrative reviews (17), these are not functionally integrated into predictive models. For clarity, molecular signatures in this review refer to genomic, transcriptomic, proteomic, or histopathological-molecular data, excluding standard clinical data such as tumor staging or routine pathology (e.g., histopathological grading) unless directly correlated with molecular profiles, which may explain the limited integration observed due to the rarity of such datasets in BSTTs. This creates a technical and conceptual disjunction: while radiomics and AI are being developed in a data-driven and task-specific manner, molecular information is often marginalized or confined to descriptive domains.

Second, within the imaging landscape, integration across different imaging modalities is pursued to varying extents. Fused imaging (e.g., PET/MRI) provides a richer phenotypic profile by integrating metabolic and anatomical data, and this has been applied successfully in soft tissue sarcoma to predict metastatic risk (22, 23). Similarly, delta-radiomics—analyzing changes in features over time—has emerged as an innovative approach for monitoring chemotherapy response (11–13). However, these approaches rarely transcend dual-modality boundaries and are themselves not combined with molecular or transcriptomic information. The resulting compartmentalization suggests a methodological segmentation of approach development. PET/MRI fusion tends to be spatially rich but biologically shallow; delta-radiomics is temporally informative but lacks molecular data integration.

Moreover, a methodological fragmentation is noticeable in how models account for anatomical, pathological, and molecular context. Certain studies attempt spatial decomposition of tumor habitats (e.g., peritumoral vs. intratumoral zone features, peritumoral edema) to achieve improved biological stratification (26, 27). Others correlate imaging changes with histopathologic endpoints such as necrosis (18). Yet a unifying framework to functionally link image-based features with biologically-derived transcriptomic or proteomic profiles is still absent. This contrasts with work in other cancer types—such as gliomas and lung cancer—where radiogenomic models are increasingly deployed to infer EGFR or IDH status directly from imaging features. The limited availability of omics-annotated datasets in BSTTs is likely a contributing factor, exacerbated by the rarity and histologic diversity of these tumors.

Importantly, the reviewed studies evidenced wide variability in methodological rigor and external validation practices. While several investigations used internal cross-validation or data splits, relatively few studies performed independent external validation (4, 26, 29). This is critical given the observed heterogeneity in imaging protocols, scanner platforms, tumor types, and feature extraction tools—variability that threatens result reproducibility without formal harmonization. Also underexplored is model explainability. While some advanced models—particularly those involving CNNs—report strong predictive performance, they often behave as black boxes that lack feature-level interpretability or clinical transparency (29). Techniques such as SHAP (Shapley Additive Explanations) or permutation importance, which are increasingly considered essential for clinical integration, are rarely incorporated in the current BSTT literature.

In summary, while notable progress has been made using radiomics and AI in BSTTs for both diagnosis and prognosis, this progress occurs largely within “two-component” silos—radiomics + AI, or imaging + pathology—with only speculative moves toward molecular integration. The integration of complete tri-modal systems (radiomics + AI + omics) remains effectively a research frontier. This absence is not due to conceptual limitations, but rather practical bottlenecks involving heterogeneous data availability, lack of matched radiomic-omic datasets, and absence of standardization across platforms. Bridging this gap will require not only multi-institutional consortia to assemble harmonized datasets but also methodological advances in feature-level and decision-level data fusion. The path to truly multi-modal BSTT intelligence systems is thus still at a formative stage—but with a clearly defined set of priorities.

### Limitations

This systematic review has several limitations, shaped both by the nature of the existing literature and the method of data retrieval. First, the scarcity of studies integrating all three components—radiomics, AI, and molecular signatures—limited the depth of analysis possible for fully integrated multi-modal approaches. Although numerous studies explored radiomics and AI together, true tri-modal integration was not found in any included studies. As a result, while the review aimed to assess synergistic multi-modal frameworks, it was compelled instead to focus on adjacent combinations.

Second, heterogeneity in tumor types, imaging protocols, and modeling techniques further complicates cross-study comparison. “Bone and soft tissue tumors” comprise a diverse group of malignancies with distinct characteristics—osteosarcoma, Ewing sarcoma, and soft tissue sarcomas were variably included across studies, with inconsistent stratification. This variability restricts the generalizability of findings and prevents meta-analytic synthesis.

Lastly, no formal risk-of-bias assessment tool (e.g., QUADAS-2 or PROBAST) was applied due to the methodological diversity of included studies. Instead, methodological quality was judged using qualitative markers (e.g., use of external validation, feature selection approach), which although informative, may not provide the standardized rigor expected in formal bias assessment.

### Suggestions for future research

To advance the integration of radiomics, AI, and molecular data in the diagnostic and prognostic modeling of BSTTs, several research directions are recommended. There is a critical need for studies that simultaneously combine imaging-derived radiomics, AI-based predictive modeling, and molecular biomarkers (e.g., gene expression, mutation panels, miRNA profiles). Public release of paired imaging-genomic datasets for BSTTs would significantly accelerate progress in this area by enabling collaborative validation and innovation.

Future research should prioritize advanced data fusion techniques—such as attention-based deep learning, graph neural networks (GNNs), or Bayesian multi-modal models—to effectively integrate heterogeneous inputs across imaging and molecular modalities. These approaches should aim to maintain biological coherence while improving predictive accuracy.

To ensure clinical applicability, studies must emphasize external validation using multi-institutional cohorts. Harmonized imaging protocols and standardized molecular data annotation will be essential to overcome current limitations in reproducibility and real-world deployment.

The adoption of explainable AI methods (e.g., SHAP, saliency mapping) should become standard practice in radiomics-AI workflows. Enhancing interpretability will be crucial for gaining clinician trust and facilitating the translation of automated systems into routine practice.

Future work should adhere to established reporting frameworks such as the Image Biomarker Standardisation Initiative (IBSI) and Radiomics Quality Score (RQS). Additionally, head-to-head comparisons of AI models using reproducible, open-source pipelines will help identify optimal methodologies.

## Conclusion

This systematic review reveals that while radiomics and artificial intelligence have made significant strides in the diagnosis and prognostication of bone and soft tissue tumors, integration with molecular signatures remains largely conceptual and unfulfilled. Current studies are segmented into radiomics-AI pipelines and pathological correlations, but no study to date unites radiomics, AI, and omics data into a single analytical model for BSTTs. Nonetheless, emerging tools such as PET/MRI fusion, delta-radiomics, and CNN-based classifiers demonstrate that multi-modal modeling is technically feasible and yields meaningful clinical insights.

## Data Availability

The original contributions presented in the study are included in the article/**Supplementary Material**. Further inquiries can be directed to the corresponding author.
