# Supplementary material for: Integrating radiomics, artificial intelligence, and molecular signatures in bone and soft tissue tumors: advances in diagnosis and prognostication
Source: Front Oncol. 2025 Aug 18;15:1613133. doi: 10.3389/fonc.2025.1613133 (PMC12399666; doi:10.3389/fonc.2025.1613133)
Supplement: Supplementary file 1 [file DataSheet1.docx]

**Supplementary File 1: Complete Search Strategy**

**Database: PubMed**

**Search Query:**

((((((((((((("Radiomics"[Mesh] OR "radiomic"[Title/Abstract] OR "delta-radiomics"[Title/Abstract] OR "texture analysis"[Title/Abstract]) AND ("Artificial Intelligence"[Mesh] OR "Machine Learning"[Mesh] OR "Deep Learning"[Mesh] OR "CNN"[Title/Abstract] OR "random forest"[Title/Abstract] OR "SVM"[Title/Abstract])) AND ("Molecular Signatures"[Title/Abstract] OR "Genomics"[Mesh] OR "Transcriptomics"[Mesh] OR "Proteomics"[Mesh] OR "multi-omics"[Title/Abstract])) AND ("Bone Neoplasms"[Mesh] OR "Soft Tissue Neoplasms"[Mesh] OR "Sarcoma"[Mesh] OR "osteosarcoma"[Title/Abstract] OR "Ewing sarcoma"[Title/Abstract] OR "liposarcoma"[Title/Abstract])) AND ("Diagnosis"[Mesh] OR "Prognosis"[Mesh] OR "Grading"[Mesh] OR "Survival Analysis"[Mesh] OR "Treatment Outcome"[Mesh])) AND ("2015/01/01"[Date - Publication] : "2025/12/31"[Date - Publication])) NOT ("Review"[Publication Type] OR "Editorial"[Publication Type] OR "Comment"[Publication Type])) NOT ("Animals"[Mesh] NOT "Humans"[Mesh])) NOT ("Case Reports"[Publication Type])) NOT ("Conference Abstract"[Publication Type]))

**Database: Scopus**

**Search Query:**

TITLE-ABS-KEY ( ( "radiomics" OR "delta-radiomics" OR "MRI radiomics" OR "CT texture" ) AND ( "artificial intelligence" OR "machine learning" OR "deep learning" OR "CNN" OR "random forest" ) AND ( "molecular signatures" OR "genomic" OR "transcriptomic" OR "proteomic" OR "multi-omics" ) AND ( "bone tumor" OR "soft tissue sarcoma" OR "osteosarcoma" OR "Ewing sarcoma" ) AND ( "diagnosis" OR "prognosis" OR "grading" OR "survival" OR "treatment response" ) ) AND PUBYEAR > 2017 AND PUBYEAR < 2026 AND ( LIMIT-TO ( DOCTYPE , "ar" ) ) AND ( EXCLUDE ( SUBJAREA , "VETE" ) OR EXCLUDE ( SUBJAREA , "AGRI" ) )

**Database: Web of Science**

**Search Query:**

TS=( ("radiomics" OR "delta-radiomics" OR "texture analysis") AND TS=( "artificial intelligence" OR "machine learning" OR "deep learning" OR "CNN" OR "support vector machine") AND TS=( "molecular signatures" OR "genomic" OR "transcriptomic" OR "proteomic") AND TS=( "bone tumor*" OR "soft tissue sarcoma" OR "osteosarcoma" OR "Ewing sarcoma") AND TS=( "diagnos*" OR "prognos*" OR "grading" OR "survival")

Refined by: [excluding] DOCUMENT TYPES: ( MEETING ABSTRACT OR BOOK CHAPTER OR EDITORIAL )

Timespan: 2015-2025

**Google Scholar**

**Search Query:**

allintitle: (radiomics OR "delta radiomics") AND ("artificial intelligence" OR "machine learning") AND ("molecular signature" OR genomics OR proteomics) AND ("bone tumor" OR "soft tissue sarcoma") AND (diagnosis OR prognosis)

Years: 2015–2025

**Keyword Combinations**

| **Category** | **Terms Used** |
| --- | --- |
| **Imaging/Radiomics** | "radiomics", "delta-radiomics", "MRI", "PET", "CT", "texture analysis" |
| **AI/ML Methods** | "machine learning", "deep learning", "CNN", "random forest", "SVM", "LASSO" |
| **Molecular Signatures** | "genomic", "transcriptomic", "proteomic", "molecular signatures", "multi-omics" |
| **Tumor Types** | "soft tissue sarcoma", "osteosarcoma", "Ewing sarcoma", "liposarcoma" |
| **Clinical Outcomes** | "diagnosis", "prognosis", "grading", "survival", "treatment response" |
