# Supplementary material for: Integrating radiomics, artificial intelligence, and molecular signatures in bone and soft tissue tumors: advances in diagnosis and prognostication
Source: Front Oncol. 2025 Aug 18;15:1613133. doi: 10.3389/fonc.2025.1613133 (PMC12399666; doi:10.3389/fonc.2025.1613133)
Supplement: Supplementary file 2 [file DataSheet2.docx]

**Supplementary File 2**

**Data Extraction Table**

| Ref | Tumor Type(s) | Sample Size (n) | Imaging Modality | Radiomics Features | AI/ML Models | Outcome | Integration of Molecular Data | Validation Strategy & Performance Metrics | Key Model Performance (best model, validation / test unless noted) |
| --- | --- | --- | --- | --- | --- | --- | --- | --- | --- |
| [1] | Soft Tissue Sarcoma (STS) | 51 patients | FDG-PET + MRI (T1, T2FS) | Texture, shape, SUV, fused PET/MRI, wavelet transform | Logistic regression | Prognostic: lung metastasis risk | None stated | Bootstrap resampling; performance specifics not listed | AUC = 0.984 for fused FDG-PET/MR texture model predicting lung metastasis 1 |
| [2] | STS | 306 patients (train 148, external test 158) | MRI (T1, T2) | Deep learning features; compared to radiomics | CNN (deep learning) | Diagnostic (tumor grading), Prognostic (mortality risk) | None stated | External validation on independent cohort; qualitative performance comparison only | AUC ��� 0.76 (T2-based DL model, independent test) 2 |
| [3] | STS | 113 patients (train 80, internal val 33) | Multiparametric MRI | Not specified beyond being “radiomics features” | Not specified; mentioned “machine learning” | Diagnostic: tumor grading | None stated | Not specified | AUC = 0.962 (RF + SMOTE model, validation) 3 |
| [4] | STS | 65 patients (train 50, test 15) | MRI (T2-weighted) | Delta-radiomics | Not specified | Prognostic: chemotherapy response | None stated | Not specified | AUC = 0.86 (RF ��-radiomics, training); test accuracy = 74.6 % (AUC not given) 4 |
| [5] | STS | ���105 patients��� (primary 70, external val ��� 35) | MRI (T1, T2FS) | Radiomics (not detailed beyond general description) | LASSO + random forest | Diagnostic: tumor grade prediction | None stated | AUC = 0.92, Accuracy = 91.43%, Sensitivity = 88.24%, Specificity = 94.44% | AUC = 0.922 (LASSO + RF, validation) 5 |
| [6] | STS | 179 patients (train 108, test 71) | MRI | Radiomics vs. semantic (radiologist-derived) features | Not specified | Prognostic: overall survival | None stated | Radiomics achieved improved risk stratification; metrics not detailed | C-index = 0.64 (T1FSGd radiomics model, test set) 6 |
| [7] | STS, Bone Sarcomas | Narrative review | MRI | Texture, signal heterogeneity | Narrative review (methods not primary) | Both Diagnostic and Prognostic | Mentions IDH and MDM2 links in literature; no new integration | N/A (Review) | N/A |
| [8] | STS | Review on AI / immunotherapy | Not specified (discusses radiomics and digital pathology) | Not specified | Review: not primary study | Both | Discussion only; potential molecular integration via immune landscape | N/A (Review) | N/A |
| [9] | STS | 30 patients | Longitudinal DWI MRI | ADC maps, delta features | SVM, logistic regression | Prognostic: therapy response (chemoradiotherapy) | None stated | AUC < 0.7 for ADC alone; other metrics not specified | AUC = 0.91 �� 0.05 (SVM with longitudinal ��-radiomics) 9 |
| [10] | Osteosarcoma | 191 patients (train 137, val 54) | CT (pre and post chemo) | Delta-radiomic features (540 extracted); robust feature selection with ICC and Pearson | LASSO + logistic regression | Prognostic: chemotherapy response | None stated | AUC used for model outcome; specific values not stated | AUC = 0.843 (��-radiomics nomogram, validation) 10 |
| [11] | BSTTs (review) | Review | MRI, CT, PET | Not detailed | Review only | General summary | Notes lack of direct radiogenomics studies; no integration performed | N/A (Review) | N/A |
| [12] | Osteosarcoma | 12 patients (102 tissue ROIs) | Multi-parametric MRI | Not specified; linked to necrosis assessment | Machine learning (not specified exact algorithm) | Prognostic: tumor necrosis post-chemotherapy | Pathology correlation with imaging | Not specified | AUC = 0.97 (mp-MRI RF classifier for necrosis) 12 |
| [13] | STS | 51 patients | PET/MRI | Texture features from three fusion methods: image-level, matrix-level, feature-level | No specific AI model named; used multivariable analysis | Prognostic: clinical outcome prediction | None stated | ROC and Mann-Whitney U tests; performance metrics not listed | AUC = 0.952 (PET/MR image-level fusion, independent validation) 13 |
| [14] | Osteosarcoma | 81 patients (train ��� 49, test ��� 32) | CT | Radiomics from CT; not detailed | Random forest (best classifier) | Prognostic: pulmonary metastasis | None stated | AUC = 0.79, Accuracy = 73% | AUC = 0.79 (RF model, independent test) 14 |
| [15] | STS (extremities/trunk) | 62 patients | MRI (fat-sat T2W) | 851 features; LASSO reduced | LASSO + Cox regression | Prognostic: disease-free survival | None stated | C-index used, DCA applied; specific values not listed | C-index = 0.781; AUC = 0.791 (combined clinical + radiomics nomogram) 15 |
| [16] | STS | 145 patients (train 102, val 43) | MRI (T1+C, T2FS) | Intratumoral habitats, peritumoral features | K-means (for habitat clustering), Logistic regression | Diagnostic: tumor grade | None stated | Multi-center (n=4); AUC reported (value not extracted) | AUC = 0.868 (habitat + peritumoral nomogram, external val) 16 |
| [17] | STS | 139 patients | MRI (with ADC) | ADC, heterogeneity, T2 and post-contrast features | Random forest | Diagnostic: malignancy grade | None stated | ADC and radiomics features statistically significant (p < 0.001); no AUC reported | AUC = 0.97; F1 = 0.93 (RF model for grade) 17 |
| [18] | STS | Review | Structural & Quant imaging (MRI, DWI, DKI, perfusion) | Not specified | Review | Diagnostic: tumor grading | None; mentions advantages/limitations | N/A (Review) | N/A |
| [19] | Osteosarcoma, Ewing Sarcoma | 66 patients | MRI (T2-FS, T1+C) | 385 features, LASSO-selected | LASSO | Diagnostic: osteosarcoma vs. Ewing sarcoma | None stated | Performance measured by AUC; value not shown | AUC = 0.881 (T2-FS model differentiating OS vs EWS) 19 |
| [20] | Osteosarcoma | 139 patients (train 60, int-val 31, time-indep 48) | DCE-MRI | Semi-quantitative (Slope, TTP, R) + radiomics | KNN, SVM, Logistic Regression | Prognostic: chemosensitivity | None stated | Performance metrics for classifiers not listed | AUC = 0.962 (validation), 0.935 (external) for MRI radiomics nomogram 20 |
| [21] | Osteosarcoma | 102 patients (train 71, test 31) | Multi-modal MRI + histopathology | Imaging features linked to histologic necrosis | Classification model (unspecified) | Prognostic: chemotherapy response | Yes – Pathologic correlation (histologic-MRI co-registration) | Not specified | AUC = 0.95 (combined radiomics + surgical stage nomogram, test) 21 |
| [22] | Osteosarcoma | 79 patients | X-ray + MRI (T1, T2, T1+C) | Not detailed; extracted from both X-ray and MRI | LASSO + logistic regression | Prognostic: response to NAC | None stated | AUC mentioned; exact numbers not listed | AUC = 0.80 (ultrasound radiomics model for high-grade STS) 22 |
| [23] | Osteosarcoma | 10 analyzable patients | FDG-PET | SUVmax, GLZLM (GLZLM-SZL) | Logistic regression | Prognostic: metastasis prediction | None stated | Cross-validation mentioned; AUC and modeling steps specified but no AUC given | Performance not quantitatively reported (pilot radiogenomic feasibility) |
| [24] | Osteosarcoma | 102 patients (train 72, val 30) | MRI (DWI) | ADC0, ADC1, DWI-based radiomics | LASSO + machine learning | Prognostic: response to chemotherapy | None stated | 70/30 training/test split; metrics not listed | AUC = 0.828 (combined clinical + X-ray + MRI radiomics, validation) 24 |

**Reference**

1. Vallières M, Freeman CR, Skamene SR, Naqa IE. A radiomics model from joint FDG-PET and MRI texture features for the prediction of lung metastases in soft-tissue sarcomas of the extremities. Phys Med Biol. 2015;60(14):5471–96.
2. Navarro F, Peeken JC, Krieg AH, Fuchs B, Ahmad SS, Müller AC, et al. Development and External Validation of Deep-Learning-Based Tumor Grading Models in Soft-Tissue Sarcoma Patients Using MR Imaging. Cancers (Basel). 2021;13(16):4092.
3. Wang H, Wang Q, Zhan X, Zhao Q, Chen Z, Li J, et al. Radiomics and Machine Learning With Multiparametric Preoperative MRI May Accurately Predict the Histopathological Grades of Soft Tissue Sarcomas. J Magn Reson Imaging. 2019;49(3):746–54.
4. Crombé A, Marcellin P, Buy X, Stoeckle E, Brouste V, Larbi A, et al. T2-based MRI Delta-radiomics improve response prediction in soft-tissue sarcomas treated by neoadjuvant chemotherapy. J Magn Reson Imaging. 2019;50(2):497–510.
5. Xu W, Wang Q, Liu P, Li X, Zhang A, Sun G, et al. Soft Tissue Sarcoma: Preoperative MRI-Based Radiomics and Machine Learning May Be Accurate Predictors of Histopathologic Grade. AJR Am J Roentgenol. 2020;215(4):963–72.
6. Peeken JC, Spraker MB, Knebel C, Dapper H, Diehl C, Gryc K, et al. Prognostic Assessment in High-Grade Soft-Tissue Sarcoma Patients: A Comparison of Semantic Image Analysis and Radiomics. Cancers (Basel). 2021;13(13):3411.
7. Fanciullo C, Colombo A, Cerri F, Cortese MC, Pianta L, Schiavi A, et al. Radiomics of Musculoskeletal Sarcomas: A Narrative Review. J Imaging. 2022;8(4):104.
8. Crombé A, Italiano A. The diagnosis, classification, and treatment of sarcoma in this era of artificial intelligence and immunotherapy. Cancer Commun (Lond). 2022;42(10):935–54.
9. Gao Y, Mccullough R, Tward DJ, O’Connor JPB, Yang Y. Treatment effect prediction for sarcoma patients treated with preoperative radiotherapy using radiomics features from longitudinal diffusion-weighted MRIs. Phys Med Biol. 2020;65(4):045006.
10. Lin P, Zhang R, Zou Q, Liu H, Li Q, Ye Z. A Delta-radiomics model for preoperative evaluation of neoadjuvant chemotherapy response in high-grade osteosarcoma. Cancer Imaging. 2020;20(1):120.
11. Zhang X, Li W, Zhang H, Huang C, Liu Y, Han Z, et al. Research status and progress of radiomics in bone and soft tissue tumors: A review. Medicine (Baltimore). 2023;102(23):e33977.
12. Huang B, Zhang S, Sun L, Zhang H, Gao Z. Feasibility of multi-parametric magnetic resonance imaging combined with machine learning in the assessment of necrosis of osteosarcoma after neoadjuvant chemotherapy: a preliminary study. BMC Cancer. 2020;20(1):881.
13. Zhao W, Liu JJ, Guo C, Wang S, Zhang T, Guo J. PET/MR fusion texture analysis for the clinical outcome prediction in soft-tissue sarcoma. Cancer Imaging. 2022;22(1):37.
14. Pereira H, Tello R, Muglia VF, Nogueira-Barbosa MH. Machine learning-based CT radiomics features for the prediction of pulmonary metastasis in osteosarcoma. Br J Radiol. 2021;94(1125):20210149.
15. Chen S, Chen Y, Lin P, Zhang R, Zou Q, Liu H, et al. Radiomics Analysis of Fat-Saturated T2-Weighted MRI Sequences for the Prediction of Prognosis in Soft Tissue Sarcoma of the Extremities and Trunk Treated With Neoadjuvant Radiotherapy. Front Oncol. 2021;11:686904.
16. Wang B, Zhang Y, Zhang Y, Meng X, Liu Y, Zhang Y, et al. Prediction of soft tissue sarcoma grading using intratumoral habitats and a peritumoral radiomics nomogram: a multi-center preliminary study. Front Oncol. 2024;14:1353225.
17. Schmitz F, Beitzel F, Quaas A, Landwehr LS, Schmeel FC, Sedaghat S. Predicting the Malignancy Grade of Soft Tissue Sarcomas on MRI Using Conventional Image Reading and Radiomics. Diagnostics (Basel). 2024;14(3):295.
18. Fang S, Liu K, Zhao X, Liang Z, Hu Z, Liu D, et al. An Update in Imaging Evaluation of Histopathological Grade of Soft Tissue Sarcomas Using Structural and Quantitative Imaging and Radiomics. J Magn Reson Imaging. 2021;54(5):1417–28.
19. Dai Y, Mu W, Qi H, Gao F, Kong L, Zhong X, et al. Differentiation of Pelvic Osteosarcoma and Ewing Sarcoma Using Radiomic Analysis Based on T2-Weighted Images and Contrast-Enhanced T1-Weighted Images. Biomed Res Int. 2020;2020:2103847.
20. Zhang L, Liu Y, Nie Y, Sun Z, Wang Z, Xia Y. Machine Learning-Based Radiomics Nomogram With Dynamic Contrast-Enhanced MRI of the Osteosarcoma for Evaluation of Efficacy of Neoadjuvant Chemotherapy. Front Oncol. 2021;11:711009.
21. Teo KY, Christopherson IS, Amyot F, Kleimeyer JP, Whitlock KB, Tomczak D, et al. Correlation of histopathology and multi-modal magnetic resonance imaging in childhood osteosarcoma: Predicting tumor response to chemotherapy. PLoS One. 2022;17(5):e0267880.
22. Luo Z, Zhao T, Liu F, Zhang Y, Gu J, Liu Y, et al. Prediction of response to preoperative neoadjuvant chemotherapy in extremity high-grade osteosarcoma using X-ray and multiparametric MRI radiomics. J Xray Sci Technol. 2023;31(3):493–507.
23. Sheen H, Kim JH, Cho YJ, Kim HS, Kang KW, Woo S. Metastasis risk prediction model in osteosarcoma using metabolic imaging phenotypes: A multivariable radiomics model. PLoS One. 2019;14(8):e0220996.
24. Zhang L, Hu Y, Zhu B, Chen D, Wang Z, Gao S. Evaluation of the neoadjuvant chemotherapy response in osteosarcoma using the MRI DWI-based machine learning radiomics nomogram. Front Oncol. 2024;14:1315984.
